# Phenolic Content and Antioxidant Activities of Burr Parsley (*Caucalis platycarpos* L.)

**DOI:** 10.3390/molecules18078666

**Published:** 2013-07-22

**Authors:** Ana Plazonić, Ana Mornar, Željan Maleš, Nikola Kujundžić

**Affiliations:** 1 Agency for Medicinal Products and Medical Devices, Ksaverska cesta 4, 10000 Zagreb, Croatia; E-Mail: ana.plazonic@halmed.hr; 2 Faculty of Pharmacy and Biochemistry, University of Zagreb, Ante Kovačića 1, 10000 Zagreb, Croatia; E-Mails: amornar@pharma.hr (A.M.); zmales@pharma.hr (Ž.M.)

**Keywords:** *Caucalis platycarpos*, activity, polyphenols

## Abstract

Since *C. platycarpos* contains a wide variety of antioxidants, in the present study total flavonoid and phenolic acid content as well as antioxidative activity of various *C. platycarpos* extracts were investigated. The results obtained show a significant polyphenol content and antioxidant activity of the investigated plant. Moreover, a positive correlation between antioxidant activity and content of flavonoids and phenolic acids was found, indicating the responsibility of these compounds for the antioxidant effectiveness of *C. platycarpos* extracts and making *C. platycarpos* a good potential source of natural antioxidants.

## 1. Introduction

Malignant diseases are progressive and, if untreated, result in death in most cases. Although certain tumors are now very amenable to treatment, malignant diseases are still one of the leading causes of death in the World. In the last decades we have witnessed huge progress in the prevention and treatment of malignant diseases. Still, discovering the causes of diseases and finding effective treatments to stop or slow their progress is an ongoing process.

Burr parsley (*Caucalis platycarpos* L.) is an annual plant growing on clayish lime-containing soils in the Mediterranean and Central Europe. In traditional herbal medicine, it has been used for the treatment of certain types of tumors. The anticancer activity of *C. platycarpos* water extract was firstly investigated using rats with artificial liver metastases of colon cancer. The rats treated by intraperitoneal injections of plant extract had significantly longer survival compared with the untreated animals. It is believed that the antitumor activity resulted from stimulation of the immunological system of the rats, by involving the spleen as a lymphatic organ to produce antitumor factors [1]. Furthermore, a more recent study has shown a significant antitumor activity of water extract of the above parts of plant in mice. The results indicated that *C. platycarpos* water extract remarkably increased the antitumor activity of the hyperthermic chemotherapy as well as the survival of mice with peritoneal carcinomatosis [2].

In order to identify the major constituents of *C. platycarpos* starch, cellulose, proteins, carbohydrates, lignin, tannins, β-sitosterol, scopoletine and flavonoids were investigated using histochemical reactions and thin-layer chromatography [3]. More recently, a selective, sensitive and accurate method using a sophisticated high-performance liquid chromatography coupled with diode-array and tandem mass spectrometric detectors (LC/DAD/MS/MS) technique was developed for the separation and identification of phenolic acids, flavonoid glycosides and aglycones in the water and methanolic extracts of *C. platycarpos*. In addition, a flavonoid glycoside, chrysoeriol-7-*O*-rutinoside, that was found to be characteristic for the investigated plant was isolated and structurally characterized by mass spectrometry. Phenolic compounds, including both phenolic acids and flavonoids, are considered to be the major bioactive compounds in *C. platycarpos* [4,5].

Since *C. platycarpos* contains a wide variety of free radical-scavenging molecules, the aim of this work was to investigate total flavonoid and phenolic acid content as well as its antioxidative activity. For the comparison of different plant extracts easy, rapid and reliable methods can be very useful, such as measuring the disappearance of colored stable free radical 2,2-diphenyl-1-picryl-hydrazyl (DPPH˙), the inhibition of membrane lipid peroxidation and xantine oxidase. Furthermore, to gain better insight into the antioxidant activity of *C. platycarpos* hydroxyl radical scavenging activity, scavenging effect on superoxide anion radical, the reducing power and the ability of plant extracts to chelate iron(II) ions were investigated.

## 2. Results and Discussion

### 2.1. Determination of Total Flavonoid Content

**Figure 1:**
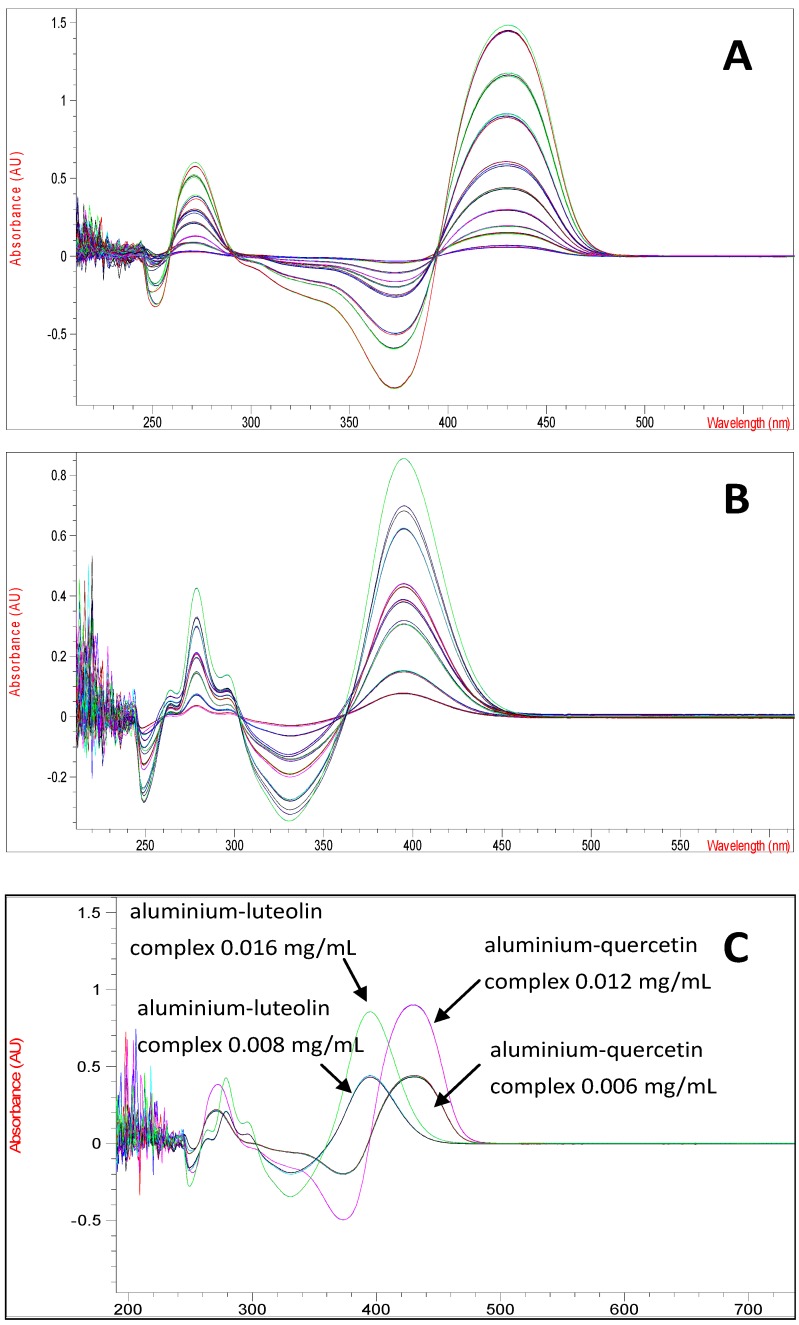
(**A**) The UV spectrum of aluminium-quercetin complex at various concentrations (0.0004–0.02 mg/mL), (**B**) the UV spectrum of aluminium-luteolin complex at various concentrations (0.0016–0.016 mg/mL) and (**C**) the UV spectrum of aluminium-quercetin complex at concentrations of 0.006 and 0.012 mg/mL and aluminium-luteolin complex at concentrations of 0.008 and 0.016 mg/mL.

The total flavonoid content of the plant extracts was determined using an optimized UV-derivative spectrophotometric method. According to our previous studies, luteolin and quercetin are the most abundant flavonoid aglycones present in *C. platycarpos* [4,5]. Unfortunately, conventional UV spectrophotometric methods for determination of total flavonoid content are unsuitable for investigation of *C. platycarpos* extracts as the aluminium-luteolin and aluminium-quercetin complexes show partial spectra overlapping. The UV spectrum of aluminium-luteolin complex has a maximum absorption at 395 nm, while aluminium-quercetin complex has its maximum absorption at 430 nm (Figure 1).

**Figure 2:**
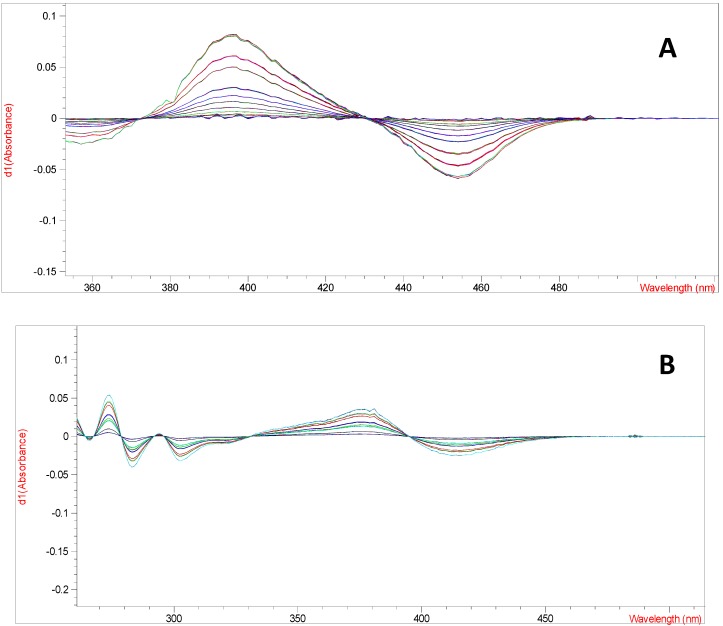
(**A**) The first-derivative spectra of aluminium-quercetin at various concentrations (0.0004–0.02 mg/mL), (**B**) and aluminium-luteolin complexes at various concentrations (0.0008–0.02 mg/mL).

Derivative spectrophotometry is an analytical technique which consists in the differentiating of normal spectrum by mathematical transformation of spectral curve into a derivative (first-or higher derivatives). It is usually used to eliminate the influence of background or matrix and provides more defined fingerprints than traditional ordinary or direct absorbance spectra, since it enhances the detectability of minor spectral features [6,7]. Therefore, an optimized UV-derivative spectrophotometric method using first-derivative spectra of luteolin and quercetin obtained after complexation with aluminium ions was used. The first-derivative spectra of aluminium-luteolin and aluminium-quercetin complexes have the maximum absorption at 372.5 and 395.0 nm, respectively (Figure 2).

**Figure 3:**
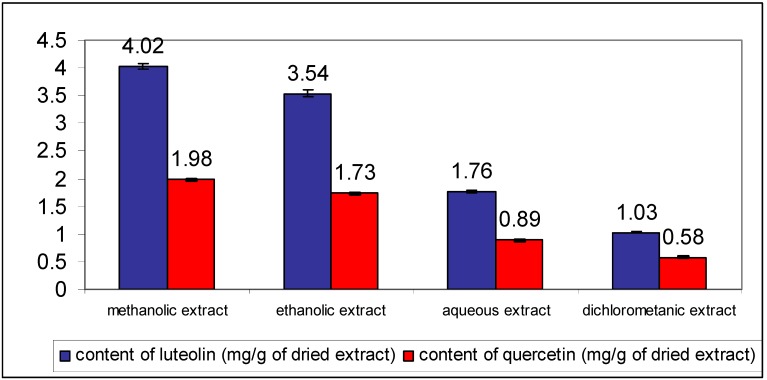
Contents of luteolin and quercetin in different *C. platycarpos* extracts. Each value is the mean ± standard deviation of six independent measurements.

To optimize the extraction process organic solvents of different polarities and water were used. The volume of the extraction solvent was increased until the complete extraction of analytes was obtained. The best extraction efficacy was obtained using methanol as extraction solvent (Figure 3). The total flavonoid content was expressed as luteolin and quercetin equivalents.

The performance characteristics of the method were evaluated with respect to linearity, precision and accuracy. The linearity of the method for determination of luteolin and quercetin was tested in the range from 0.8 to 20 μg/mL. At least, seven concentration levels were used in both calibration curves. The calibration curves were obtained at 372.5 nm and 395.0 nm for luteolin and quercetin, respectively. The obtained correlation coefficients were higher than 0.997 indicating satisfactory linearity of developed method. The detection (LOD) and the quantification (LOQ) limits were established from the calibration curve as LOD = 3 s/b and LOQ = 10 s/b, where s is the residual standard deviation of the calibration curve and b is the slope of the calibration curve. The LOD of luteolin was found to be 0.35 μg/mL, while the LOQ was 1.05 μg/mL. The LOD and LOQ values of quercetin were a slightly lower, 0.26 and 0.77 μg/mL, respectively. The precision of the method was investigated using three different concentrations of luteolin (1.4, 6.4 and 12.0 μg/mL) and quercetin (2.0, 6.2 and 12.4 μg/mL). Each concentration level was analyzed individually five times. The inter-day precision was assessed by three replicate analyses on three consecutive days. The results of precision measurements, expressed as relative standard deviations (RSDs), are summarized in Table 1. The RSD values for intra- and inter-day precision were lower than 1.95 and 2.87%, respectively. Furthermore, the accuracy of the method was investigated using three different known concentrations of luteolin (1.6, 3.2 and 8.0 μg/mL) and quercetin (2.0, 4.0 and 8.0 μg/mL). The results of accuracy measurements, expressed as recoveries, are listed in Table 1. The recoveries of both analytes were in range from 92.37 to 98.33%. These results indicate the satisfactory accuracy of the method.

**Table 1:** Validation data of total flavonoids determination.

*Validation parameter*	*Luteolin*	*Quercetin*
Linearity range (μg/mL)	0.8–20	0.8–20
Equation	y = 0.090 − 0.001	y = 0.161 − 0.001
Calibration coefficient (*r*^2^)	0.997	0.999
Limit of detection (μg/mL)	0.35	0.26
Limit of quantitation (μg/mL)	1.05	0.77
Precision (RSD, %)		
Intra-day		
Low	1.79	1.37
Medium	0.88	1.35
High	1.95	1.34
Inter-day		
Low	2.60	1.75
Medium	2.87	2.36
High	1.86	2.08
Accuracy (Recovery, %)		
Low	92.37	94.83
Medium	94.07	94.01
High	98.33	97.08

### 2.2. Determination of Total Phenolic Acids Content

The total phenolic acid content was determined using a UV spectrophotometric technique. According to our previous studies, the most abundant phenolic acid after hydrolysis of phenolic acid esters in *C. platycarpos* extracts was chlorogenic acid [4,5]. Therefore, the method optimization and validation was performed using chlorogenic acid as well as the total phenolic acid content was expressed as chlorogenic acid equivalents.

**Figure 4:**
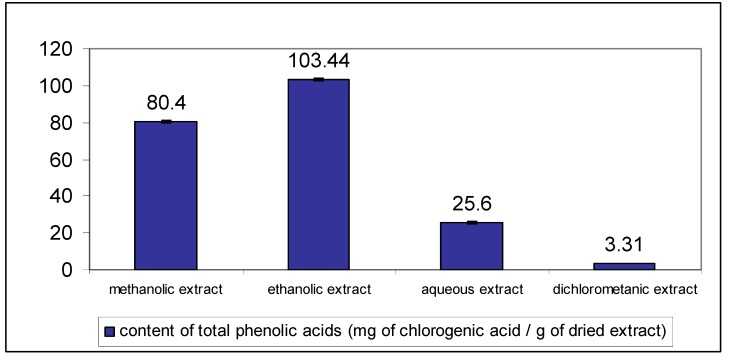
Contents of total phenolic acids expressed as milligrams of chlorogenic acid per gram of dried extract in different *C. platycarpos* extracts. Each value is the mean ± standard deviation of six independent measurements.

To optimize the extraction process organic solvents of different polarities and water were used. The extracts contained from 3.31 to 103.44 mg/g phenolic acids expressed as chlorogenic acid equivalents. The best extraction efficacy was obtained using ethanol (*v/v*) (Figure 4).

The validation of the method was performed and following parameters were tested: linearity, precision and accuracy. The linearity of the method for determination of chlorogenic acid was tested in the range from 0.01 to 0.50 mg/mL. The calibration curve was linear over concentration range studied (*r*^2^ = 0.999) and the slope of the equation was 1.5818 while the intercept was 0.0125. The LOD and LOQ values, established from the calibration curve, were 0.003 and 0.010 mg/mL. The precision of the method was investigated using three different concentrations of luteolin (0.05, 0.10 and 0.40 mg/mL). The procedure was repeated five times within the same day to obtain the intra-day precision while the inter-day precision was assessed by replicate analysis on three continual days. The results of precision measurements, expressed as RSDs, are reported in Table 2.

The RSD values of intra- and inter-day precision were less than 1.69%, indicating excellent precision of the method. Furthermore, the accuracy of the method was investigated using three different known concentrations of chlorogenic acid (0.05, 0.10 and 0.30 mg/mL). The accuracy of measurements was expressed in terms of recovery (Table 2).

**Table 2:** Validation data of total phenolic acids determination.

*Validation parameter*	*Chlorogenic acid*
Linearity range (mg/mL)	0.010–0.500
Equation	y = 1.582x + 0.013
Calibration coefficient (*r*^2^)	0.999
Limit of detection (mg/mL)	0.003
Limit of quantitation (mg/mL)	0.010
Precision (RSD, %)	
Intra-day	
Low	1.61
Medium	1.57
High	0.94
Inter-day	
Low	1.69
Medium	1.67
High	1.47
Accuracy (Recovery, %)	
Low	99.00
Medium	100.97
High	98.27

### 2.3. Determination of Antioxidative Activity

In the last few decades the antioxidant activity of plant samples has been the subject of numerous *in vitro* and *in vivo* studies. Antioxidative activity has become the one of the most interesting biological properties of naturally occurring substances present in higher plants. Beneficial health effects of an array of herbal preparations have been ascribed to the antioxidant activity of plants, and in such way as to prevent in the development of various diseases, including tumors [8,9,10,11,12].

To the best of our knowledge, there are no literature data concerning the antioxidant properties of *C. platycarpos* extracts. Therefore, the antioxidant properties of plant were evaluated in order to find a new natural source of antioxidants. Since the antioxidant compounds found in plants have different polarities, different solvents were used to isolate antioxidants. Methanol, ethanol, dichloromethane and water are the most commonly used solvents in extraction processes. The antioxidant activity of the extract and yield depends strongly on the used solvent. In order to get a better insight in the antioxidant activity of *C. platycarpos* extracts different assays were tested.

Firstly, the free-radical-scavenging activity of different plant extracts was tested using the DPPH method. The DPPH radical is a commonly used substrate for fast evaluation of antioxidant activity because of its stability in the radical form and simplicity of the assay. The principle behind this assay is the color change of DPPH solution from purple to yellow as the radical quenched by an antioxidant. The results, expressed as IC_50_ values, are presented in Table 3. and it should be pointed out that a lower IC_50_ value indicates a greater antioxidant activity. The IC_50_ values obtained by the DPPH method for different plant extracts ranged from 42.6 to 2,690 μg/mL. The obtained results indicate that most of free radical-scavenging molecules were extracted using methanol as extraction solvent.

The inhibitory effect on lipid peroxidation of plant extracts were evaluated using bovine brain phospholipid liposomes as model membranes. Protection against free radical-induced lipid peroxidation by the *C. platycarpos* extracts may be of great importance for plant traditional use against inflammatory diseases, many of which are associated with membrane damage and tissue injury. Lipid peroxidation was stimulated with ascorbic acid, well known antioxidant. Still, ascorbic acid at concentrations up to 10^−3^ M shows prooxidant properties. Fe^3+^ and, to a lesser extent Co^2+^, act synergistically with ascorbic acid. Moreover, the prooxidant effect increases with metal concentration. The results, expressed as IC_50_ values, are summarized in Table 3. The IC_50_ values obtained for different *C. platycarpos* extracts ranged from 0.47 to 2.50 mg/mL. The obtained results indicate that the highest amounts of compounds, inhibitors of membrane lipid peroxidation, are present in methanolic and ethanolic extracts.

The antioxidant activity of prepared plant extracts was also evaluated using the deoxyribose assay. Hydroxyl radicals, generated by reaction of an iron-EDTA complex with H_2_O_2_ in the presence of ascorbic acid, attack deoxyribose to form products that, upon heating with thiobarbituric acid, yield a pink chromogen. Added hydroxyl radical “scavengers” compete with deoxyribose for the hydroxyl radicals produced and diminish chromogen formation. The results, expressed as IC_50_ values, are reported in Table 3. The highest antioxidative activity was obtained for ethanolic extract. Slightly higher IC_50_ values were obtained for methanolic and aqueous extracts.

As it was found that some polyphenolic compounds show an inhibitory effect on xantine oxidase, inhibition of xantine oxidase activity by different *C. platycarpos* extracts was investigated. Xanthine oxidase is a form of xanthine oxidoreductase, an enzyme that catalyzes the oxidation of hypoxanthine to xanthine and can further catalyze the oxidation of xanthine to uric acid. During the oxidation process hydrogen peroxide and superoxide anion are produced. These active oxygen species have been proposed to cause oxidative injury to tissues, cell death as well as DNA damage which causes mutation and chromosomal damage. Xanthine oxidase activity was determined by quantifying the amount of uric acid produced from xanthine in the raection mixture by spectrophotometry. Unfortunately, *C. platycarpos* extract did not show significant effect on xantine oxidase activity.

Superoxide dismutase (SOD) is one of the most important antioxidative enzymes which catalyzes the dismutation of the superoxide anion into hydrogen peroxide and molecular oxygen. Several direct and indirect methods have been developed to determine SOD activity. An indirect method using nitrotetrazolium blue is often used because of its convenience. Still, there are several disadvantages to this method, such as poor water solubility of the formazan dye and its reaction with the reduced form of xanthine oxidase. Therefore, a SOD Assay Kit-WST was employed. This method allowed a very convenient and highly sensitive determination of SOD activity by utilizing highly water-soluble tetrazolium salt, WST-1, which produces a water-soluble formazan dye upon reduction with a superoxide anion. The rate of WST-1 reduction by superoxide anion is linearly related to the xanthine oxidase activity and is inhibited by SOD. The scavenging effect of the *C. platycarpos* extracts on superoxide anion radical, expressed as IC_50_ values are presented in Table 3. While the significant scavenging effect was obtained by methanolic, ethanolic and aqueous extracts, extract prepared with dichloromethane showed little or no scavenging effect on the free radicals.

**Table 3:** Comparative overview of antioxidant activity of *C. platycarpos* extracts obtained by various assays.

*Sample*	*Methanolic extract*	*Ethanolic extract*	*Aqueous extract*	*Dichloromethanic extract*	*Positive control*
DPPH scavenging activity (μg/mL)	42.6 ± 8.9	61.9 ± 3.5	95.9 ± 12.7	2690 ± 130	2.4 ± 0.3
Inhibition of membrane lipid peroxidation (μg/mL)	470 ± 30	530 ± 30	2240 ± 290	2500 ± 250	4.6 ± 0.2
OH˙ scavenging activity (μg/mL)	10.2 ± 0.8	5.2 ± 1.3	12.1 ± 1.1	23.3 ± 3.0	0.4 ± 0.1
Inhibition of xantine oxidase	nd	nd	nd	nd	nd
Superoxideanion radical scavenging activity (μg/mL)	320 ± 20	280 ± 20	500 ± 30	nd	nt
Reducing power (μmol/g)	108.3 ± 9.0	146.6 ± 7.6	82.4 ± 6.0	31.7 ± 1.0	5.68 ± 0.20
Ion chelating activity (μg/mL)	370 ± 60	400 ± 60	1240 ± 80	nd	185.0 ± 2.0

nd: not determined at tested concentrations; nt: not tested.

Reducing power is associated with antioxidant activity, therefore may serve as a significant reflection of the antioxidant activity of compound as well as plant extract. Compounds with reducing power indicate that they are electron donors and can reduce the oxidized intermediates of lipid peroxidation processes, so that they can act as primary and secondary antioxidants. In this assay, the yellow color of the test solutions changed to various shades of green and blue depending on the reducing power of each sample. Presence of reducers caused the conversion of the ferricyanide complex to the ferrous form. By measuring formation of Pearl’s Prussian blue at 700 nm, it is possible to determine the concentration of Fe^+3^ ions. All investigated plant extracts have shown high reducing power (Table 3). Still, the highest reducing power was found for ethanolic extract. Furthermore, the increased reducing power with increased concentration was observed for all extracts. High reducing power of investigated extracts suggested remarkable potency of plant constituents to donate electrons to reactive free radicals, thus converting them into more stable non-reactive species and finally terminate the free radical chain reaction.

The antioxidant activity of *C. platycarpos* extracts are also attributed to the ability of its constituents to chelate transition metal ions, such as iron, which has been proposed as the catalyst for the initial formation of reactive oxygen species. Furthermore, iron(II) ion is known as a potent inducer of lipid peroxidation as it accelerates lipid oxidation by breaking down hydrogen and lipid peroxides to reactive free radicals via the Fenton-type reaction. Ferrozine can quantitatively form complexes with iron(II) ions. This complex formation can be disrupted in the presence of chelating agents resulting in a decrease in the red color of the complex. The metal chelating activity of chelator can be successfully evaluated by measuring the color intensity reduction at the 562 nm wavelength. Methanolic, ethanolic and aqueous *C. platycarpos* extracts demonstrated an ability to chelate iron(II) ions (Table 3). No distinctive difference between the IC_50_ values of methanolic and ethanolic extracts was obtained, while value obtained for aqueous extract was significantly higher. As mentioned above, EDTA was used as a positive control as it is one of the most powerful metal chelator ever known. Unfortunately, it should be pointed out that the chelating abilities of all *C. platycarpos* extracts were much lower than EDTA.

Finally, the influence of total flavonid and phenolic acids content on antioxidant activities of *C. platycarpos* extracts was evaluated. The results showed very positive correlation between amounts of flavonoids and phenolic acids and antioxidative activities determined in following assays: inhibition of membrane lipid peroxidation (*r* = 0.9700 − 0.9871), superoxide dismutase activity (*r* = 0.9216 − 0.9936) and metal ion chelating (*r* = 0.9471 − 0.9856). Furthermore, good correlation was obtained between amounts of phenolic acids and antioxidative activities determined in the deoxyribose and reducing power assays (*r* = 0.8970 − 0.9587). No significant correlation between amounts of flavonoids and phenolic acids and antioxidative activities determined was in DPPH assays.

## 3. Experimental

### 3.1. Plant material and Extraction Procedure

The aboveground parts of the plant *C. platycarpos*, were collected in the surroundings of Imotski, Croatia, in June 2008, identified by Professor Nikola Kujundžić, one of the authors. The voucher specimens have been deposited in the Department of Analytical Chemistry, Faculty of Pharmacy and Biochemistry, University of Zagreb, Croatia. The plant material was air dried, smashed into power and stored in a dessicator.

The extraction of air dried and grounded, aboveground parts of plant material were performed using different solvents: methanol, ethanol (96%), dichloromethane and water. About 1 g of accurately weight herb material was extracted with 10 mL of solvent at room temperature, on a magnetic stirrer (3,000 rpm) for 14 h. Filtered extracts were evaporated under reduced pressure at 60 °C to dryness and stored in a refrigerator at −20 °C until use. Just before use dried extracts were dispersed in methanol.

### 3.2. Chemicals and Instruments

2,2’-Diphenyl-1-picrylhydrazyl, disodium EDTA dihydrate, xanthine-oxidase from buttermilk and luteolin were purchased from Fluka BioChemika (Buchs, Switzerland). 2-Deoxy-d-ribose, ferrozine, phosphate buffer (pH = 7.4), ferrous chloride tetrahydrate, brain extract Type VII from Bovine Brain, butylhydroxytolunene, quercetin (HPLC grade) as well as superoxide-dismutase Assay Kit were from Sigma-Aldrich (Steinheim, Germany). Acetic acid, aluminium chloride hexahydrate, dichloromethane, disodium hydrogen phosphate dihydrate, ethanol, ethylacetate, methanol, potassium hexacyanoferrate, sodium dihydrogen phosphate dihydrate, sodium hydroxide, sodium molybdate dihydrate, sodium nitrite, thiobarbituric acid, trichloroacetic acid of analysis quality were obtained by Kemika (Zagreb, Croatia). L(+)- Ascorbic acid together with ferric chloride anhydrous were purchased from Riedel-de Haën (Buchs, Switzerland) while hydrochloric acid 35% G.R. was obtained by Lach-Ner (Neratovice, Czech Republic). 3-*O*-caffeoylquinic acid and rutin, both HPLC grade, were from Acros Organics (Geel, Belgium). Ferric ammonium sulphate was obtained by J. T. Baker (Philipsburg, NJ, USA) while xanthine was from Sigma (St Louis, MO, USA). Analyses were carried out using an Agilent UV-VIS 8453 diode array spectrophotometer (Agilent Technologies, Waldbronn, Germany), thermostated water bath (INKO, Zagreb, Croatia), magnetic stirrer (Thermolyne Cimarec 2, Barnstead, Dubuque, IA, USA) and microplate reader (Labsystems iEMS Reader MF, Helsinki, Finland).

### 3.3. Phytochemical Analysis of Polyphenols

#### 3.3.1. Determination of Total Flavonoid Content

##### 3.3.1.1. Preparation of Standard Solutions

The stock solution of luteolin and quercetin (0.2 mg/mL) were prepared by dissolving with methanol. All working standard solutions were prepared transferring appropriate amounts of the stock solutions to 25 mL volumetric task following by addition of 10 mL ethyl acetate, 0.5 mL 0.5% aqueous solution of sodium citrate and 2 mL of aluminium chloride.

##### 3.3.1.2. Preparation of Samples

Each extract (10.00 mL) was transferred to a 25.00 mL calibrated flask following by addition of aqueous solution of sodium citrate (0.5 mL, 0.5%) and aluminium chloride (2 mL, 2 g of alluminium chloride hexahidrate in 100 mL 5% methanolic solution of acetic acid). Finally, the flask was filled up to the mark by a mixture of methanol and acetic acid (95:5, *v/v*). The samples were allowed to stand at room temperature for 45 min. Determination of flavonoid content was performed using UV/VIS spectrophotometric technique. The analysis was performed according to the Christ and Müller method with small variations [13,14].

Values for first-derivative spectra for luteolin at 372.5 nm and for quercetin at 395.0 nm were recorded. The same mixture without aluminium chloride was used as reference solution. All measurements were performed in triplicate. Validation of the method was performed following the recommendation of International Conference on Harmonization (ICH) [15].

#### 3.3.2. Determination of Total Phenolic Acids Content

##### 3.3.2.1. Preparation of Standard Solutions

The stock solution of chlorogenic acid (1 mg/mL) was prepared by dissolving with 50% ethanol (*v/v*). Working standard solutions (0.01–0.50 mg/mL) used for method optimization were prepared immediately before measurements by mixing suitable aliquots of the stock solution and ethanol.

##### 3.3.2.2. Preparation of samples

Extract (1.00 mL) was transferred into a 15.00 mL calibrated flask, followed by addition of hydrochloric acid (2 mL, 0.5 M), sodium nitrite (2 mL, 100 mg/mL), sodium molybdate (2 mL, 100 mg/mL) and sodium hydroxide solution (2 mL, 85 mg/mL). The calibrated flask was filled up to the mark with ultrapure water. The content of total phenolic acids was performed according to the European Pharmacopoeia spectrometric method with slight modifications [16]. The absorbance of the complex formed between phenolic acid and sodium nitrite–sodium molybdate was measured using UV/VIS spectrophotometric technique at 530 nm. The same mixture without sodium nitrite and sodium molybdate was used as referent solution. All measurements were performed in triplicate. Validation of the method was performed according to ICH quidelines [15].

### 3.4. Evaluation of Antioxidative Activity

#### 3.4.1. DPPH method

Free radical scavenging activities of the plant extracts at concentrations in the range from 1 to 3,500 μg/mL were determined in accordance with the Molyneux [17] method, which is based on the principle of scavenging the DPPH radical. The methanolic solution (0.2 mM) of DPPH was added into the solutions prepared with various plant extracts of different concentrations (final concentration 2–3500 μg/mL) and stirred. Each mixture was kept in dark at room temperature for 30 min and the absorbance was measured at 517 nm against a blank. All measurments were performed in triplicate. The percentage of the antioxidative activity was calculated using the following equation:

Antioxidative activity (%) = ((A_0_ − A_1_)/A_0_) × 100 (1)

where A_0_ is the absorbance of the control and A_1_ is the absorbance in the presence of the plant extract. The actual decrease in absorption induced by the test was compared with the positive controls, the solution of rutin (0.66–5.94 μg/mL). The IC_50_ (concentration of plant extract providing 50% inhibition) values were calculated using the dose inhibition curve in linear range by plotting the extract concentration versus the corresponding scavenging effect.

#### 3.4.2. Inhibition of Membrane Lipid Peroxidation

Inhibition of membrane lipid peroxidation by plant extracts was determined by the method of Houghtonu and co-workers [18] with some slight modifications. Briefly, phospholipid liposomes were prepared by suspension of bovine brain extract in potassium phosphate buffer (pH 7.4). The peroxidation of bovine brain’s phospholipids was induced by addition of Fe^3+^-ascorbate. Plant extract (300 μL) was added to bovine brain extract (500 μL). Afterwards, the samples were incubated for 30 min at 37 °C. Thiobarbituric acid (1 mL, 1%) in NaOH (0.05 M) and etanolic solution of butylated hydroxytoluene (100 μL, 2%) were added to the incubation mixture and incubated was continued for 20 minat 100 °C. The thiobarbituric acid-reactive species were extracted with butanol and quantified by spectrophotometry at 532 nm. All experiments were run in triplicate. The percentage of the antioxidative activity was calculated using Equation (1). The actual decrease in absorption induced by the test was compared with the positive controls, the solution of quercetin. The IC_50_ (concentration of plant extract providing 50% inhibition) values were calculated using the dose inhibition curve in linear range by plotting the extract concentration versus the corresponding percentage of inhibition of membrane lipid peroxidation.

#### 3.4.3. The Deoxyribose Method

The assay was performed as described by Halliwell and co-workers [19] with minor changes. The hydroxyl radical scavenging activity of plant extracts was determined by incubating the following reagents at the final concentration in a volume of 1 mL for 1 h at 37 °C: deoxyribose (100 μL, 2.8 mM) and plant extracts (500 μL) in phosphate buffer (pH 7.4), EDTA (200 μL, 0.1 mM), FeCl3 (200 μL, 0.05 mM), H2O2 (100 μL, 1.0 mM) and ascorbate (100 μL, 1.0 mM). After addition of trichloroacetic acid (1 mL, 2.8%) and thiobarbituric acid (1 mL, 1%), the solutions were heated at 100 °C for 20 min. After cooling the absorbance of samples was measured at 532 nm against a blank. All assays were carried out in triplicate. The percentage of the antioxidative activity was calculated using Equation (1). The actual decrease in absorption induced by the test was compared with the positive controls, the solution of rutin (0.03–6.60 μg/mL). The IC_50_ (concentration of plant extract providing 50% inhibition) values were calculated using the dose inhibition curve in nonlinear range by plotting the extract concentration versus the corresponding percentage of the hydroxyl radical scavenging activity.

#### 3.4.4. Inhibition of Xantine Oxidase Activity

Assay principle for determination of inhibition of xantine oxidase activity by plant extracts is based on the method of Ahmad and co-workers with some modifications [20]. The samples were prepared by mixing xanthine (800 μL, 0.15 mM) dissolved in deionized water, EDTA (200 μL, 0.1 mM) and plant extracts (1000 μL). The reaction was catalyzed by xantine oxidase solution (1000 μL, prepared fresh, 10 mU/mL) in phosphate buffer (pH 7.4, 200 mM). The assay mixture was incubated at 37 °C for 30 min. Before measuring the absorbance at 290 nm, the reaction was stopped by adding HCl (300 µL, 0.58 M). Absorbance was measured spectrophotometrically against a blank prepared in the same way but replacing xanthine oxidase with phosphate buffer. Alopurinol was used as a positive control. All assays were run in triplicate. Percent inhibition of xantine oxidase activity was calculated by comparison with control group using the following equation:

Inhibition of xantine oxidase activity (%) = A_1_/A_2_ × 100 (2)

where A_1_ is the absorbance in the presence of the plant extract and A_2_ is the absorbance of the control.

#### 3.4.5. Superoxide Anion Radical Scavenging Assay

The scavenging effect of the plant extracts on superoxide anion radical was measured using SOD Assay Kit-WST. The kit includes WST-1 (2-(4-iodophenyl)-3-(4-nitrophenyl)-5-(2,4-disulfo-phenyl)-2H-tetrazolium, monosodium salt) solution, buffer solution, enzyme solution and dilution buffer. Plant extract (20 μL) was placed into each well of a microplate. Afterwards, WST working solution (200 μL) and enzyme working solution (20 μL) were added to the well. The plate was incubated for 20 min at 37 °C. Since the absorbance at 440 nm is proportional to the amount of superoxide anion, the SOD activity was quantified by measuring the decrease in the color development at 440 nm. The scavenging effect or inhibition rate was calculated using following equation:

SOD activity ={[(A_blank1_ − A_blank3_) − (A_sample_ − A_blank2_)]/(A_blank1_ − A_blank3_)}×100 (3)

where A_sample_ is the absorbance in the presence of the plant extract, A_blank1_ is the absorbance of sample solution where plant extract was replaced with ultrapure water, A_blank2_ is the absorbance of sample solution where enzyme working solution with replaced by dilution buffer and A_blank3_ is the absorbance of sample solution where plant extract and enzyme working solution were replaced with ultrapure water and dilution buffer. All assays were carried out in triplicate. The IC_50_ values were calculated using the dose inhibition curve in linear range by plotting the extract concentration versus the corresponding percentage of inhibition rate.

#### 3.4.6. Reducing Power Assay

The reducing power of plant extracts was evaluated by adopting the procedure described by Oyaizu [21]. The plant extracts (500 μL, 2–500 μg/mL) were suspended in phosphate buffer (1 mL, pH 6.6, 0.2 M) and potassium hexacyanoferrate (1 mL, 1%). The mixture was incubated at 50 °C for 20 min, after which trichloroacetic acid (1 mL) was added to the mixture. After centrifugation at 1,800 rpm for 10 min, the supernatant (1 mL) was mixed with an equal amount of ultrapure water and FeCl_3_ (200 μL, 0.1%). The absorbance of the obtained solution was measured at 700 nm. Solution of ascorbic acid (0.1 mM) prepared at the same manner was used as a positive control. Assays were carried out in triplicate. The reducing power of plant extracts were expressed as Ascorbic Acid Equivalents (AscAE) units per gram of dried extract according to following equation:

AscAE (μmol/g of dried extract) = (c × A_1_ × 10^6^)/(γ × A_2_) × 100 (4)

where c is the concentration of ascorbic acid (mM), A_1_ is the absorbance in the presence of the plant extract, γ is the concentration of sample (mg/L) and A_2_ is the absorbance of positive control.

#### 3.4.7. Metal Ion Chelating Assay

The ability of samples to chelate iron(II) ions was evaluated using the method reported by Carter [22] with some modifications and compared with that of the reference chelator agent EDTA. Plant extracts (400 μL, 2–1,700 μg/mL) were added to solution of iron(II) chloride (200 μL, 2 mM) and methanol (1800 μL). The reaction was initiated by addition of ferrozine (800 μL, 5 mM). The reaction mixture was shaken vigorously and left standing at room temperature for 10 min. Afterwards, the absorbance of the solution was measured spectrophotometrically at 562 nm. EDTA was used as a positive control. All assays were done in triplicate. The percentage of ferrozine-Fe^2+^ complex formation was calculated using Equation (1). The IC_50_ (concentration of plant extract providing 50% complex formation) values were calculated by plotting the extract concentration versus the corresponding percentage of ferrozine-Fe^2+^ complex formation.

## 4. Conclusions

In this study the total flavonoid and phenolic acid content as well as antioxidative properties of various *C. platycarpos* extracts were determined. The results obtained show a significant polyphenol content and antioxidant activity of the investigated plant. Generally, a positive relationship between antioxidant activities and phenolic content was observed. Although further work is required, we may presume that *C. platycarpos* possesses good medicinal potential.
